# Herpesviruses in Captive Chelonians in Europe Between 2016 and 2020

**DOI:** 10.3389/fvets.2021.733299

**Published:** 2021-10-13

**Authors:** Christoph Leineweber, Elisabeth Müller, Rachel E. Marschang

**Affiliations:** Laboklin GmbH & Co. KG, Bad Kissingen, Germany

**Keywords:** Emydidae, season, testudinid herpesvirus, Testudinidae, *Testudo*, *Terrapene*, tortoise, turtle

## Abstract

Herpesviruses are important pathogens in tortoises and turtles, yet little is known about the epidemiology of these viruses. We analyzed herpesviruses detected by PCR in samples from captive chelonians in Europe according to virus strain, host species, year and season in which the animal was tested, and country in which the animal was kept. A total of 4,797 samples submitted to a diagnostic laboratory in Europe between January 2016 and December 2020 were evaluated. Of these, 312 (6.50%) were positive for herpesviruses. The types most commonly found were testudinid herpesvirus (TeHV)1 (143 positive, 45.83%) and TeHV3 (153 positive, 49.04%), but also included TeHV2 (1 positive, 0.32%), TeHV4 (3 positive, 0.96%), Terrapene herpesvirus 1 (7 positive, 2.24%), Trachemys herpesvirus 1 (2 positive, 0.64%), and three previously undescribed herpesviruses (0.96%). Herpesviruses were detected in chelonians in the families Testudinidae, Emydidae, Geoemydidae, and in the suborder Pleurodira. Among the species for which 100 samples or more were available, the highest proportions of positive samples (positivity rates) were found in samples from Horsfield's tortoises (*Testudo horsfieldii*) (14.96%), and radiated tortoises (*Astrochelys radiata*) (14.05%). Among tortoises (Testudinidae), viruses were most often detected in the spring, while in emydid turtles (Emydidae) they were most often detected in the summer. A comparison of the positivity rates according to country showed significant differences, with the highest rate in samples from Italy (16.01%). This study indicated possible differences in herpesvirus positivity rates depending on host species, virus strain, year of sampling, season, and country of origin. It provides useful information in further understanding fluctuations in infection rates as well as in helping to guide decision making for herpesvirus diagnostics in chelonian patients. It also provides evidence for the international dispersal of herpesviruses with their hosts through international trade.

## Introduction

Herpesviruses are among the most commonly described viruses in chelonians. Herpesviruses found in these animals that have been genetically analyzed have clustered together in the subfamily *Alphaherpesvirinae*, in the genus *Scutavirus* (1–3). That genus currently contains two species: *Chelonid alphaherpevirus 5* and *Testudinid alphaherpesvirus 3* (4). Additional herpesviruses have been described in multiple species of chelonian and have been shown to cause significant disease in many cases.

Herpesviruses found in sea turtles (family Cheloniidae) include *Chelonid alphaherpesvirus 5* which is associated with fibropapillomatosis (3, 5, 6). Lung-eye-trachea-disease-associated virus (LETV) was originally described in green turtles (*Chelonia mydas*) with respiratory disease (7). It is categorized in the species *Chelonid alphaherpesvirus 6* (4), and is related to the members of the genus *Scutavirus*, but has not been assigned to a genus yet. Other herpesviruses described in sea turtles include gray patch disease virus found in juvenile green turtles with skin lesions (8). No information on the genome of that virus is available. Loggerhead genital-respiratory herpesvirus (LGRV) and loggerhead orocutaneous herpesvirus (LOCV) were both found in wild-caught loggerhead turtles (*Caretta caretta*). Analysis of sequences from a part of the DNA-dependent DNA-polymerase gene showed that both clustered with members of the genus *Scutavirus* (9).

A number of herpesviruses have been described in tortoises in the family Testudinidae. The best characterized of these is *Testudinid alphaherpesvirus 3* (TeHV3), the genome of which has been fully sequenced (2, 10). Some genetic diversity has been found in members of this species (2, 10). Transmission studies with TeHV3 isolates in spur-thighed tortoises (*Testudo graeca*) and Hermann's tortoises (*T. hermanni*) have demonstrated a connection between infection and disease, including apathy, anorexia, nasal discharge and oral lesions (2, 11). TeHV3 has been shown to infect a wide range of testudinid tortoise species (3). Several other genetically distinct herpesviruses have also been described in tortoises. These have commonly been referred to as testudinid herpesvirus 1, 2, and 4 (TeHV1, 2, and 4). These have all been less well-investigated than TeHV3. TeHV1 was originally described in Japan in imported pancake (*Malacochersus tornieri*) and Horsfield's (*Testud horsfieldii*) tortoises (12). This virus has been most frequently found in Horsfield's tortoises in Europe, although it can also infect other tortoise species (13). TeHV2 was first described in a captive desert tortoise (*Gopherus agassizii*) in California, USA (14). TeHV4 was found by chance during a quarantine examination of a clinically healthy Bowsprit tortoise (*Chersina angulata*) at a zoo in the USA (15). It has also been found in a leopard tortoise (*Stigmochelys pardalis*) in Europe (16). Reported prevalence of testudinid herpesviruses in diagnostic samples from captive tortoises has ranged between 8.2 and 25% (13, 17–20).

A variety of herpesviruses have also been detected in wild and captive turtles of other families in recent years. Most of these reports have been from animal in the families Emydidae and Geoemydidae. While reports of herpesvirus infections in these species were originally based on histological and electronmicroscopic findings (21–23), recent detections have been based more often on PCR and sequencing. These viruses have been divided into different strains based on partial sequencing data and have generally been designated based on the host species in which they were detected. Emydid herpesvirus 1 has been detected in several species of emydid turtles, including northern map turtles (*Graptemys geographica*), painted turtles (*Chrysemys picta*), and eastern river cooters (*Pseudemys concinna concinna*) in the United States and Germany (24, 25) as well as in a mixed collection including *Trachemys* spp., *Graptemys* spp., and *Pseudemys* spp. in Germany (3). Emydid herpesvirus 2 was detected in asymptomatic bog turtles (*Glyptemys muhlenbergii*) and spotted turtles (*Clemmys guttata*) (26). Glyptemys herpesvirus 1 and glyptemys herpesvirus 2 were both detected in asymptomatic bog and wood turtles (*Glyptemys insculpta*) (26). Trachemys herpesvirus 1 has been detected in asymptomatic free ranging red-eared sliders (*Trachemys scripta elegans*) in the United States (27). Emydoidea herpesvirus 1 was detected in free ranging Blanding's turtles (*Emydoidea blandingii*) in the United States (28), while Emydoidea herpesvirus 2 was detected in a squamous cell carcinoma in the oral cavity of a free ranging Blanding's turtle (29). Terrapene herpesvirus 1 and 2 have each been described in Eastern box turtles (*Terrapene carolina carolina*). Terrapene herpesvirus 1 has been reported associated with disease outbreaks in captive box turtles (30, 31). It has also been detected at high prevalence among free ranging eastern box turtles in the United States (32). Terrapene HV2 was detected in an eastern box turtle with recurrent papillomatous skin lesions (33).

Herpesviruses have also been detected in turtles in several other families in individual cases. Chelydra herpesvirus 1 was found in free ranging common snapping turtles (*Chelydra serpentina*) in the United States (27). Pelomedusid herpesvirus 1 was detected in clinically healthy captive West African mud turtles (*Pelusios castaneus*) in Europe (34). Another, unnamed herpesvirus was reported in a William's mud turtle (*Pelusios williamsi*) with papillomatous skin lesions in Europe (35).

Molecular detection and differentiation of herpesviruses in chelonians has most often been carried out using a pan-herpesvirus PCR targeting a conserved portion of the DNA-dependent DNA-polymerase gene (36). In some cases, additional PCRs have been developed targeting specific chelonid herpesviruses, e.g., for TeHV1 and 3 (11–13, 37, 38). Real-time PCRs have been described for the detection of several strains of herpesviruses found in emydid turtles (28, 39, 40).

There is only limited data available on the seasonality of herpesvirus infections in turtles and tortoises. In surveys of free ranging eastern box turtles for a variety of pathogens, Terrapene HV 1 has been detected significantly more often in summer (40) or fall (32) than in other seasons. Season has been hypothesized to play a role in disease development and virus shedding in tortoises (13, 41). However, no long term studies of patterns of herpesvirus infection are currently available.

The purpose of this study was to analyse detection of herpesviruses in samples from chelonians submitted to a diagnostic laboratory in Europe. It was hypothesized that herpesvirus detection would differ depending on herpesvirus type, season, and year and that the type of herpesvirus detected would depend on the host species. In addition, the country of origin of the sample was hypothesized to impact the prevalence of herpesvirus detection.

## Materials and Methods

Samples submitted to Laboklin GmbH & Co. KG (Bad Kissingen, Germany), a veterinary diagnostic laboratory, over the course of a 5 year period (2016–2020) were evaluated. Samples were submitted by veterinarians and pet owners. Only samples identified as having been collected from a chelonian were included in the study, although the host species was not provided in all cases. The vast majority of samples tested were oral swabs. Reasons for testing were not provided, and background information on the animals was not generally available. No samples were solicited for this study. Repeat or multiple samples from individual animals were counted as single samples.

All samples were processed within 24 h of arrival at the laboratory. DNA was extracted using a commercial kit (MagNA Pure 96 DNA and viral NA small volume kit, Roche, Penzberg, Germany) according to the manufacturer's instructions. Three different PCRs were performed for the detection of herpesviral DNA as described previously (13). These included a consensus PCR targeting a small region of the DNA-dependent DNA-polymerase gene that is able to detect a wide range of herpesviruses in the family *Herpesviridae* (36), a second PCR targeting a portion of the DNA polymerase gene able to detect TeHV1 (12, 38), and a PCR targeting a part of the *UL5* gene able to detect TeHV3 (37, 38). For all samples in which only one of these PCRs was positive or in which a herpesvirus or the herpesvirus type identified had not been previously reported in that host species, the PCR product was sequenced by Sanger sequencing. In these cases, PCR products were purified (MinElute purification kit, Qiagen, Hilden, Germany) according to the manufacturer's instructions. Sequencing was performed using a Big-Dye Terminator v3.1 cycle sequencing kit (Life Technologies, Carlsbad, CA, USA) and analyzed on an ABI 3130 sequencer (Applied Biosystems, Waltham, MA, USA). Sequences were manually edited and primer sequences were removed for further analysis. Sequences were compared to those in GenBank using BLAST (https://blast.ncbi.nlm.nih.gov/Blast.cgi).

Statistical analyses were carried out using the statistical analysis software (SAS) (SAS Institute, Cary, NC, USA) for the calculation of the proportion of positive samples, refered to as positivity rates in this text. The 95% binomial confidence intervals were calculated based on the Wilson procedure (42). The Pearson chi-squared test was used with a type I error α of 0.05 to test the independence of compared positivity rates. Fisher exact test was used for small sample sizes for the specific calculations on Testudinidae and Emydidae. A *p*-value <0.05 was considered significant.

## Results

### Herpesviruses Detected

From the beginning of 2016 to the end of 2020 4,797 samples from chelonians were tested for herpesviruses by PCR. Overall, 312 (6.50%; 95% CI 5.84–7.23%) were positive for herpesviruses. Of these positive samples, 143 were positive for TeHV1 (45.83%; 95% CI 40.39–51.38%), one for TeHV2 (0.32%; 95% CI 0.06–1.79%), 153 for TeHV3 (49.04%; 95% CI 43.54–54.56%), 3 for TeHV4 (0.96%; 95% CI 0.33–2.79%), 7 for Terrapene herpesvirus 1 (2.24%; 95% CI 1.09–4.55%), 2 for Trachemys herpesvirus 1 (0.64%; 95% CI 0.18–2.31%), and 3 for other alphaherpesviruses (0.96%; 95% CI 0.33–2.79%) (Table 1). Of the 3 that were positive for other alphaherpesviruses, one was from an oral swab from a Siebenrock's snake-necked turtle (*Chelodina rugosa*) collected in Italy in March 2018. The sequence obtained from that sample was 100% identical to that from a herpesvirus found in a panther chameleon (*Furcifer pardalis*) (GenBank accession No. MW015088.1) and did not cluster with members of the *Scutavirus* genus (43). One was from an oral swab from a Chinese striped-necked turtle (*Mauremys sinensis*) collected in France in August 2019. The sequence obtained from that sample had the highest identity (76.4%) to a herpesvirus detected in a Williams' mud turtle (*Pelusios williamsi*) (GenBank accession No. KX374559.1) and clustered with other herpesviruses from chelonians in the *Scutavirus* genus. The third was from an oral swab from a European pond turtle (*Emys orbicularis*) collected in Italy in November 2020. The sequence from this sample had the highest identity (94.4%) to Terrapene herpesvirus 1 (GenBank accession No. KJ004665.1). The sequences from the TeHV2, TeHV4, and other alphaherpesviruses not described previously in chelonians are provided in Supplementary Table 1.

**Table 1:** Chelonian species tested and herpesvirus positivity rate depending on species and virus strain.

**Family**	**Species**	**Total (*n*)**	**HV positive**	**TeHV 1**	**TeHV 2**	**TeHV 3**	**TeHV 4**	**Terrapene HV 1**	**Trachemys herpesvirus 1**	**Other alphaherpes** **viruses**
Testudinidae	Tortoise	363	14 (3.86%; CI 2.31–6.37%)	6 (1.65%; CI 0.76–3.55%)	0 (0%; CI 0–1.05%)	8 (2.20%; CI 1.12–4.28%)	0 (0%; CI 0–1.05%)	0 (0%; CI 0–1.05%)	0 (0%; CI 0–1.05%)	0 (0%; CI 0–1.05%)
	*Testudo* spp.	33	8 (24.24%; CI 12.83–41.02%)	2 (6.06%; CI 1.68–19.61%)	0 (0%; CI 0–10.43%)	6 (18.18%; CI 8.61–34.39%)	0 (0%; CI 0–10.43%)	0 (0%; CI 0–10.43%)	0 (0%; CI 0–10.43%)	0 (0%; CI 0–10.43%)
	*Testudo hermanni*	1,072	50 (4.66%; CI 3.55–6.09%	7 (0.65%; CI 0.31–1.34%)	0 (0%; CI 0–0.36%)	43 (4.01%; CI 2.99–5.36%)	0 (0%; CI 0–0.36%)	0 (0%; CI 0–0.36%)	0 (0%; CI 0–0.36%)	0 (0%; CI 0–0.36%)
	*Testudo graeca*	464	29 (6.25%; CI 4.39–8.83%)	4 (0.86%; CI 0.33–2.19%)	0 (0%; CI 0–0.82%)	25 (5.39%; CI 3.68–7.84%)	0 (0%; CI 0–0.82%)	0 (0%; CI 0–0.82%)	0 (0%; CI 0–0.82%)	0 (0%; CI 0–0.82%)
	*Testudo marginata*	171	10 (5.85%; CI 3.21–10.43%)	3 (1.75%; CI 0.60–5.02%)	0 (0%; CI 0–2.20%)	7 (4.09%; CI 1.99–8.20%)	0 (0%; CI 0–2.20%)	0 (0%; CI 0–2.20%)	0 (0%; CI 0–2.20%)	0 (0%; CI 0–2.20%)
	*Testudo horsfieldii*	361	54 (14.96%; CI 11.65–19.01%)	53 (14.68%; CI 11.40–18.70%)	0 (0%; CI 0–1.05%)	1 (0.28%; CI 0.05–1.56%)	0 (0%; CI 0–1.05%)	0 (0%; CI 0–1.05%)	0 (0%; CI 0–1.05%)	0 (0%; CI 0–1.05%)
	*Testudo kleinmani*	51	2 (3.92%; CI 1.08–13.21%	2 (3.92%; CI 1.08–13.21%)	0 (0%; CI 0–0.70%)	0 (0%; CI 0–0.70%)	0 (0%; CI 0–0.70%)	0 (0%; CI 0–0.70%)	0 (0%; CI 0–0.70%)	0 (0%; CI 0–0.70%)
	*Centrochelys sulcata*	181	6 (3.31%; CI 1.52–7.4%)	5 (2.76%; CI 1.18–6.30%)	0 (0%; CI 0–2.08%)	1 (0.55%; CI 0.10–3.06%)	0 (0%; CI 0–2.08%)	0 (0%; CI 0–2.08%)	0 (0%; CI 0–2.08%)	0 (0%; CI 0–2.08%)
	*Astrochelys radiata*	121	17 (14.05%; CI 8.96–21.35%)	0 (0%; CI 0–3.08%)	0 (0%; CI 0–3.08%)	17 (14.05%; CI 8.96–21.35%)	0 (0%; CI 0–3.08%)	0 (0%; CI 0–3.08%)	0 (0%; CI 0–3.08%)	0 (0%; CI 0–3.08%)
	*Stigmochelys pardalis*	231	13 (5.63%; CI 3.32–9.39%)	6 (2.60%; CI 1.20–5.55%)	0 (0%; CI 0–1.64%)	5 (2.16%; CI 0.93–4.96%)	2 (0.87%; CI 0.24–3.11%)	0 (0%; CI 0–1.64%)	0 (0%; CI 0–1.64%)	0 (0%; CI 0–1.64%)
	*Aldabrachelys gigantea*	98	0 (0%; CI 0–3.77%)	0 (0%; CI 0–3.77%)	0 (0%; CI 0–3.77%)	0 (0%; CI 0–3.77%)	0 (0%; CI 0–3.77%)	0 (0%; CI 0–3.77%)	0 (0%; CI 0–3.77%)	0 (0%; CI 0–3.77%)
	*Chelonoidis nigra*	26	0 (0%; CI 0–12.87%)	0 (0%; CI 0–12.87%)	0 (0%; CI 0–12.87%)	0 (0%; CI 0–12.87%)	0 (0%; CI 0–12.87%)	0 (0%; CI 0–12.87%)	0 (0%; CI 0–12.87%)	0 (0%; CI 0–12.87%)
	*Chelonoidis carbonarius*	86	1 (1.16%; CI 0.20–6.29%)	0 (0%; CI 0–4.28%)	0 (0%; CI 0–4.28%)	1 (1.16%; CI 0.20–6.29%)	0 (0%; CI 0–4.28%)	0 (0%; CI 0–4.28%)	0 (0%; CI 0–4.28%)	0 (0%; CI 0–4.28%)
	*Chelonoidis chilensis*	35	10 (28.57%; CI 16.33–45.05%)	9 (25.71%; CI 14.16–42.06%)	0 (0%; CI 0–9.89%)	1 (2.86%; CI 0.51–14.54%)	0 (0%; CI 0–9.89%)	0 (0%; CI 0–9.89%)	0 (0%; CI 0–9.89%)	0 (0%; CI 0–9.89%)
	*Chelonoidis denticulata*	14	0 (0%; CI 0–21.53%)	0 (0%; CI 0–21.53%)	0 (0%; CI 0–21.53%)	0 (0%; CI 0–21.53%)	0 (0%; CI 0–21.53%)	0 (0%; CI 0–21.53%)	0 (0%; CI 0–21.53%)	0 (0%; CI 0–21.53%)
	*Geochelone elegans*	73	1 (1.37%; CI 0.24–7.36%)	1 (1.37%; CI 0.24–7.36%)	0 (0.0%; CI 0–5.0%)	0 (0%; CI 0–5.0%)	0 (0%; CI 0–5.0%)	0 (0%; CI 0–5.0%)	0 (0%; CI 0–5.0%)	0 (0%; CI 0–5.0%)
	*Kinixys sp*.	3	0 (0%; CI 0–56.15%)	0 (0%; CI 0–56.15%)	0 (0%; CI 0–56.15%)	0 (0%; CI 0–56.15%)	0 (0%; CI 0–56.15%)	0 (0%; CI 0–56.15%)	0 (0%; CI 0–56.15%)	0 (0%; CI 0–56.15%)
	*Indotestudo elongata*	12	0 (0%; CI 0–24.25%)	0 (0%; CI 0–24.25%)	0 (0%; CI 0–24.25%)	0 (0%; CI 0–24.25%)	0 (0%; CI 0–24.25%)	0 (0%; CI 0–24.25%)	0 (0%; CI 0–24.25%)	0 (0%; CI 0–24.25%)
	*Gopherus berlandien*	8	1 (12.50%; CI 2.24–47.09%)	0 (0%; CI 0–32.44%)	1 (12.50%; CI 2.24–47.09%)	0 (0%; CI 0–32.44%)	0 (0%; CI 0–32.44%)	0 (0%; CI 0–32.44%)	0 (0%; CI 0–32.44%)	0 (0%; CI 0–32.44%)
	*Malacochersus tornieri*	14	0 (0%; CI 0–21.53%)	0 (0%; CI 0–21.53%)	0 (0%; CI 0–21.53%)	0 (0%; CI 0–21.53%)	0 (0%; CI 0–21.53%)	0 (0%; CI 0–21.53%)	0 (0%; CI 0–21.53%)	0 (0%; CI 0–21.53%)
	*Homopus* ssp.	11	0 (0%; CI 0–25.88%)	0 (0%; CI 0–25.88%)	0 (0%; CI 0–25.88%)	0 (0%; CI 0–25.88%)	0 (0%; 0–25.88%)	0 (0%; CI 0–25.88%)	0 (0%; CI 0–25.88%)	0 (0%; CI 0–25.88%)
	*Manouria* spp.	13	0 (0%; CI 0–22.81%)	0 (0%; CI 0–22.81%)	0 (0%; CI 0–22.81%)	0 (0%; CI 0–22.81%)	0 (0%; CI 0–22.81%)	0 (0%; CI 0–22.81%)	0 (0%; CI 0–22.81%)	0 (0%; CI 0–22.81%)
	*Pyxis* spp.	19	0 (0% CI 0–16.82%)	0 (0%; CI 0–16.82%)	0 (0%; CI 0–16.82%)	0 (0%; CI 0–16.82%)	0 (0%; CI 0–16.82%)	0 (0%; CI 0–16.82%)	0 (0%: CI 0–16.82%)	0 (0%; CI 0–16.82%)
	*Geochelone platynota*	6	0 (0%; CI 0–39.03%)	0 (0%; CI 0–39.03%)	0 (0%; CI 0–39.03%)	0 (0%; CI 0–39.03%)	0 (0%; CI 0–39.03%)	0 (0%; CI 0–39.03%)	0 (0%; CI 0–39.03%)	0 (0%; CI 0–39.03%)
	*Psammobates* spp.	4	0 (0%; CI 0–48.99%)	0 (0%; CI 0–48.99%)	0 (0%; CI 0–48.99%)	0 (0%; CI 0–48.99%)	0 (0%; CI 0–48.99%)	0 (0%; CI 0–48.99%)	0 (0%; CI 0–48.99%)	0 (0%; CI 0–48.99%)
	*Astrochelys yniphora*	2	0 (0%; CI 0–65.76%)	0 (0%; CI 0–65.76%)	0 (0%; CI 0–65.76%)	0 (0%; CI 0–65.76%)	0 (0%; CI 0–65.76%)	0 (0%; CI 0–65.76%)	0 (0%; CI 0–65.76%)	0 (0%; CI 0–65.76%)
	*Chersina angulata*	1	0 (0%; CI 0–79.35%)	0 (0%; CI 0–79.35%)	0 (0%; CI 0–79.35%)	0 (0%; CI 0–79.35%)	0 (0%; CI 0–79.35%)	0 (0%; CI 0–79.35%)	0 (0%; CI 0–79.35%)	0 (0%; CI 0–79.35%)
	*Chersobius signatus*	2	0 (0%; CI 0–65.76%)	0 (0%; CI 0–65.76%)	0 (0%; CI 0–65.76%)	0 (0%; CI 0–65.76%)	0 (0%; CI 0–65.76%)	0 (0%; CI 0–65.76%)	0 (0%; CI 0–65.76%)	0 (0%; CI 0–65.76%)
Emydidae	Turtles	16	0 (0%; CI 0–19.36%)	0 (0%; CI 0–19.36%)	0 (0%; CI 0–19.36%)	0 (0%; CI 0–19.36%)	0 (0%; CI 0–19.36%)	0 (0%; CI 0–19.36%)	0 (0%; CI 0–19.36%)	0 (0%; CI 0–19.36%)
	*Emys orbicularis*	33	1 (3.03%; CI 0.54–15.32%)	0 (0%; CI 0–10.43%)	0 (0%; CI 0–10.43%)	0 (0%; CI 0–10.43%)	0 (0%; CI 0–10.43%)	0 (0%; CI 0–10.43%)	0 (0%; CI 0–10.43%)	1 (3.03%; CI 0.54–15.32%)
	*Trachemys* spp.	18	0 (0%; CI 0–17.59%)	0 (0%; CI 0–17.59%)	0 (0%; CI 0–17.59%)	0 (0%; CI 0–17.59%)	0 (0%; CI 0–17.59%)	0 (0%; CI 0–17.59%)	0 (0%; CI 17.59%)	0 (0%; CI 0–17.59%)
	*Trachemy scripta elegans*	48	1 (2.08%; CI 0.37–10.89%	0 (0%; CI 0–7.41%)	0 (0%; CI 0–7.41%)	0 (0%; CI 0–7.41%)	0 (0%; CI 0–7.41%)	0 (0%; CI 0–7.41%)	1 (2.08%; CI 0.37–10.89%)	0 (0%; CI 0–7.41%)
	*Trachemys scripta scripta*	19	1 (5.26%; CI 0.93–24.63%)	0 (0%; CI 0–16.82%)	0 (0%; CI 0–16.82%)	0 (0%; CI 0–16.82%)	0 (0%; CI 0–16.82%)	0 (0%; CI 0–16.82%)	1 (5.26%; CI 0.93–24.63%)	0 (0%; CI 0–16.82%)
	*Pseudemys* spp.	2	0 (0%; CI 0–65.76%)	0 (0%; CI 0–65.76%)	0 (0%; CI 0–65.76%)	0 (0%; CI 0–65.76%)	0 (0%; CI 0–65.76%)	0 (0%; CI 0–65.76%)	0 (0%; CI 0–65.76%)	0 (0%; CI 0–65.76%)
	*Graptemys* spp.	7	0 (0%; CI 0–35.43%)	0 (0%; CI 0–35.43%)	0 (0%; CI 0–35.43%)	0 (0%; CI 0–35.43%)	0 (0%; CI 0–35.43%)	0 (0%; CI 0–35.43%)	0 (0%; CI 0–35.43%)	0 (0%; CI 0–35.43%)
	*Terrapene* spp.	30	7 (23.33%; CI 11.79–40.92%)	0 (0%; CI 0–11.35%)	0 (0%; CI 0–11.35%)	0 (0%; CI 0–11.35%)	0 (0%; CI 0–11.35%)	7 (23.33%; CI 11.79–40.92%)	0 (0%; CI 0–11.35%)	0 (0%; CI 0–11.35%)
	*Chrysemys* spp.	2	0 (0%; CI 0–65.76%)	0 (0%; CI 0–65.76%)	0 (0%; CI 0–65.76%)	0 (0%; CI 0–65.76%)	0 (0%; CI 0–65.76%)	0 (0%; CI 0–65.76%)	0 (0%; CI 0–65.76%)	0 (0%; CI 0–65.76%)
	*Clemmys guttata*	1	0 (0%; CI 0–79.35%)	0 (0%; CI 0–79.35%)	0 (0%; CI 0–79.35%)	0 (0%; CI 0–79.35%)	0 (0%; CI 0–79.35%)	0 (0%; CI 0–79.35%)	0 (0%; CI 0–79.35%)	0 (0%; CI 0–79.35%)
Geoemydidae	*Mauremys* spp.	15	1 (6.67%; CI 1.19–29.82%)	0 (0%; CI 0–20.39%)	0 (0%; CI 0–20.39%)	0 (0%; CI 0–20.39%)	0 (0%; CI 0–20.39%)	0 (0%; CI 0–20.39%)	0 (0%; CI 0–20.39%)	1 (6.67%; CI 1.19–29.82%)
	*Geoemyda spengleri*	5	0 (0%; CI 0–43.45%)	0 (0%; CI 0–43.45%)	0 (0%; CI 0–43.45%)	0 (0%; CI 0–43.45%)	0 (0%; CI 0–43.45%)	0 (0%; CI 0–43.45%)	0 (0%; CI 0–43.45%)	0 (0%; CI 0–43.45%)
	*Batagur affinis*	4	0 (0%; CI 0–48.99%)	0 (0%; CI 0–48.99%)	0 (0%; CI 0–48.99%)	0 (0%; CI 0–48.99%)	0 (0%; CI 0–48.99%)	0 (0%; CI 0–48.99%)	0 (0%; CI 0–48.99%)	0 (0%; CI 0–48.99%)
	*Curora* spp.	11	0 (0%; CI 0–25.88%)	0 (0%; CI 0–25.88%)	0 (0%; CI 0–25.88%)	0 (0%; CI 0–25.88%)	0 (0%; CI 0–25.88%)	0 (0%; CI 0–25.88%)	0 (0%; CI 0–25.88%)	0 (0%; CI 0–25.88%)
	*Rhinoclemmys pucherrima*	10	0 (0%; CI 0–27.75%)	0 (0%; CI 0–27.75%)	0 (0%; CI 0–27.75%)	0 (0%; CI 0–27.75%)	0 (0%; CI 0–27.75%)	0 (0%; CI 0–27.75%)	0 (0%; CI 0–27.75%)	0 (0%; CI 0–27.75%)
	*Heosemys grandis*	2	0 (0%; CI 0–65.76%)	0 (0%; CI 0–65.76%)	0 (0%; CI 0–65.76%)	0 (0%; CI 0–65.76%)	0 (0%; CI 0–65.76%)	0 (0%; CI 0–65.76%)	0 (0%; CI 0–65.76%)	0 (0%; CI 0–65.76%)
	*Orlitia borneensis*	2	0 (0%; CI 0–65.76%)	0 (0%; CI 0–65.76%)	0 (0%; CI 0–65.76%)	0 (0%; CI 0–65.76%)	0 (0%; CI 0–65.76%)	0 (0%; CI 0–65.76%)	0 (0%; CI 0–65.76%)	0 (0%; CI 0–65.76%)
Cheloniidae	Sea turtle	10	0 (0%; CI 0–27.75%)	0 (0%; CI 0–27.75%)	0 (0%; CI 0–27.75%)	0 (0%; CI 0–27.75%)	0 (0%; CI 0–27.75%)	0 (0%; CI 0–27.75%)	0 (0%; CI 0–27.75%)	0 (0%; CI 0–27.75%)
Kinostenidae	*Kinosternon leucostomum*	4	0 (0%; CI 0–48.99%)	0 (0%; CI 0–48.99%)	0 (0%; CI 0–48.99%)	0 (0%; CI 0–48.99%)	0 (0%; CI 0–48.99%)	0 (0%; CI 0–48.99%)	0 (0%; CI 0–48.99%)	0 (0%; CI 0–48.99%)
	*Sternotherus* spp.	4	0 (0%; CI 0–48.99%)	0 (0%; CI 0–48.99%)	0 (0%; CI 0–48.99%)	0 (0%; CI 0–48.99%)	0 (0%; CI 0–48.99%)	0 (0%; CI 0–48.99%)	0 (0%; CI 0–48.99%)	0 (0%; CI 0–48.99%)
Platysternidae	*Platysternon megacephalum*	4	0 (0%; CI 0–48.99%)	0 (0%; CI 0–48.99%)	0 (0%; CI 0–48.99%)	0 (0%; CI 0–48.99%)	0 (0%; CI 0–48.99%)	0 (0%; CI 0–48.99%)	0 (0%; CI 0–48.99%)	0 (0%; CI 0–48.99%)
Carettochelydidae	*Carettochelys insculpta*	2	0 (0%; CI 0–65.76%)	0 (0%; CI 0–65.76%)	0 (0%; CI 0–65.76%)	0 (0%; CI 0–65.76%)	0 (0%; CI 0–65.76%)	0 (0%; CI 0–65.76%)	0 (0%; CI 0–65.76%)	0 (0%; CI 0–65.76%)
Chelydridae	*Chelydra serpentina*	3	0 (0%; CI 0–56.15%)	0 (0%; CI 0–56.15%)	0 (0%; CI 0–56.15%)	0 (0%; CI 0–56.15%)	0 (0%; CI 0–56.15%)	0 (0%; CI 0–56.15%)	0 (0%; CI 0–56.15%)	0 (0%; CI 0–56.15%)
Suborder Pleurodira	*Podocnemis* ssp.	3	0 (0%; CI 0–56.15%)	0 (0%; CI 0–56.15%)	0 (0%; CI 0–56.15%)	0 (0%; CI 0–56.15%)	0 (0%; CI 0–56.15%)	0 (0%; CI 0–56.15%)	0 (0%; CI 0–56.15%)	0 (0%; CI 0–56.15%)
	*Phrynops hilarii*	1	0 (0%; CI 0–79.35%)	0 (0%; CI 0–79.35%)	0 (0%; CI 0–79.35%)	0 (0%; CI 0–79.35%)	0 (0%; CI 0–79.35%)	0 (0%; CI 0–79.35%)	0 (0%; CI 0–79.3%)	0 (0%; CI 0–79.35%)
	*Chelodina longicollis*	1	1 (100%; CI 20.65–100%)	0 (0%; CI 0–79.35%)	0 (0%; CI 0–79.35%)	0 (0%; CI 0–79.35%)	0 (0%; CI 0–79.35%)	0 (0%; CI 0–79.35%)	0 (0%; CI 0–79.35%)	1 (100%; CI 20.65–100%)
	*Chelus fimbriata*	3	0 (0%; CI 0–56.15%)	0 (0%; CI 0–56.15%)	0 (0%; CI 0–56.15%)	0 (0%; CI 0–56.15%)	0 (0%; CI 0–56.15%)	0 (0%; CI 0–56.15%)	0 (0%; CI 0–56.15%)	0 (0%; CI 0–56.15%)
Unknown Family	Unknown Species	1,062	84 (7.91%; CI 6.43–9.69%)	45 (4.24%; CI 3.18–5.63%)	0 (0%; CI 0–0.36%)	38 (3.58%; CI 2.62–4.88%)	1 (0.09%; CI 0.02–0.52%)	0 (0%; CI 0–0.36%)	0 (0%; CI 0–0.36%)	0 (0%; CI 0–0.36%)

*Results shown as: Number positive [% positive of total tested from each individual species; 95% confidence interval (CI)]*.

### Herpesvirus Detection According to Host Family, Year of Sampling, and Country of Origin

The vast majority of samples tested were from tortoises in the family Testudinidae (3,475 samples), 216 of these (6.22%; 95% CI 5.46–7.07%) were positive for herpesviruses. In the other families tested, 10 of 176 samples from Emydidae were positive (5.68%; 95% CI 3.11–10.14%) and no significant (*p* = 0.7743) difference between the positivity rates were noted between these two families. One sample (2.04%; 95% CI 0.36–10.69%) from the family Geoemydidae (*n* = 49) and one (12.50%; 95% CI 2.24–47.09%) from the suborder Pleurodira (*n* = 8) were positive. All other samples from Cheloniidae (*n* = 10), Kinosternidae (*n* = 8), Platysternidae (*n* = 4), Carettochelyidae (*n* = 2), and Chelydridae (*n* = 3) tested were negative. 1,062 samples could not be assigned a specific family, 84 of these were tested positive (7.91%; CI 6.43–9.69%) (Table 1). A comparison between the different tested species showed significant (*p* < 0.0001) differences.

The herpes virus positivity rates varied significantly (*p* = 0.0002) between 4.78 and 9.47% depending on the year of sampling (Table 2). A significant variation (*p* < 0.0001) was also found between the seasons in which the samples were submitted, with the highest positivity rate in spring (9.48%) and the lowest in fall (2.98%) (Table 3).

**Table 2:** Herpesvirus positivity rate according to virus strain and year of sampling.

**Year**	**Total (*n*)**	**Herpesvirus positive**	**TeHV 1**	**TeHV 2**	**TeHV 3**	**TeHV 4**	**Terrapene HV 1**	**Trachemys herpesvirus 1**	**Other alphaherpes viruses**
2016	681	58 (8.52%; CI 6.65–10.86%)	31 (4.55% CI 3.22–6.39%)	0 (0%; CI 0–0.56%)	22 (3.24%; CI 2.14–4.84%)	2 (0.29%; CI 0.08–1.06%)	1 (0.15%; CI 0.03–0.83%)	2 (0.29%; CI 0.08–1.06%)	0 (0%, CI 0–0.56%)
2017	581	55 (9.47%; CI 7.35–12.12%)	25 (4.30%; CI 2.93–6.27%)	1 (0.17%; CI 0.03–0.96%)	28 (4.82%; CI 3.36–6.88%)	0 (0%; CI 0–0.66%)	1 (0.17%; CI 0.03–0.96%)	0 (0%; CI 0–0.66%)	0 (0%; CI 0–0.66%)
2018	1,178	68 (5.77%; CI 4.58–7.25%)	32 (2.72%; CI 1.93–3.81%)	0 (0%; CI 0–0.33%)	29 (2.46%; CI 1.72–3.51%)	1 (0.08%; CI 0.01–0.47%)	5 (0.42%; CI 0.18–0.98%)	0 (0%; CI 0–0.33%)	1 (0.08%; CI 0.01–0.47%)
2019	1,235	59 (4.78%; CI 3.72–6.12%)	27 (2.19%; CI 1.51–3.17%)	0 (0%; CI 0–0.31%)	31 (2.51%; CI 1.77–3.54%)	0 (0%; CI 0–0.31%)	0 (0%; CI 0–0.31%)	0 (0%; CI 0–0.31%)	1 (0.08%; CI 0.01–0.46%)
2020	1,122	72 (6.42%; CI 5.13–8.01%)	28 (2.50%; CI 1.74–3.59%)	0 (0%; CI 0–0.34%)	43 (3.83%; CI 2.86–5.12%)	0 (0%; CI 0–0.34%)	0 (0%; CI 0–0.34%)	0 (0%; CI 0–0.34%)	1 (0.09%; CI 0.02–0.50%)

*Results shown as: Number positive [% positive out of total tested in that year; 95% confidence interval (CI)]*.

**Table 3:** Herpesvirus positivity rate according to virus strains and season of sampling.

**Season**	**Total (*n*)**	**Herpesvirus positive**	**TeHV 1**	**TeHV 2**	**TeHV 3**	**TeHV 4**	**Terrapene HV 1**	**Trachemys herpesvirus 1**	**Other alphaherpes viruses**
Spring	1,666	158 (9.48%; CI 8.17–10.98%)	90 (5.40%; CI 4.41–6.59%)	0 (0%; CI 0–0.23%)	66 (3.96%; CI 3.12–5.01%)	1 (0.06%; CI 0.01–0.34%)	0 (0%; CI 0–0.23%)	0 (0%; CI 0–0.23%)	1 (0.06%; CI 0.01–0.34%)
Summer	1,368	78 (5.70%; CI 4.59–7.06%)	23 (1.68%; CI 1.12–2.51%)	0 (0%; CI 0–0.28%)	49 (3.58%; CI 2.72–4.70%)	0 (0%; CI 0–0.28%)	5 (0.37%; CI 0.16–0.86%)	0 (0%; CI 0–0.28%)	1 (0.07%; CI 0.01–0.41%)
Fall	1,075	32 (2.98%; CI 2.12–4.18%)	12 (1.12%; CI 0.64–1.95%)	1 (0.09%; CI 0.02–0.52%)	15 (1.40%; CI 0.85–2.30%)	0 (0%; CI 0–0.36%)	1 (0.09%; CI 0.02–0.52%)	2 (0.19%; CI 0.05–0.68%)	1 (0.09%; CI 0.02–0.52%)
Winter	688	44 (6.40%; CI 4.80–8.48%)	18 (2.62%; CI 1.66–4.10%)	0 (0%; CI 0–0.56%)	23 (3.34%; CI 2.24–4.96%)	2 (0.29%; CI 0.08–1.05%)	1 (0.15%; CI 0.03–0.83%)	0 (0%; CI 0–0.56%)	0 (0%; CI 0–0.56%)

*Results shown as: Number positive [% positive out of total tested in each season; 95% confidence interval (CI)]*.

**Figure 1:**
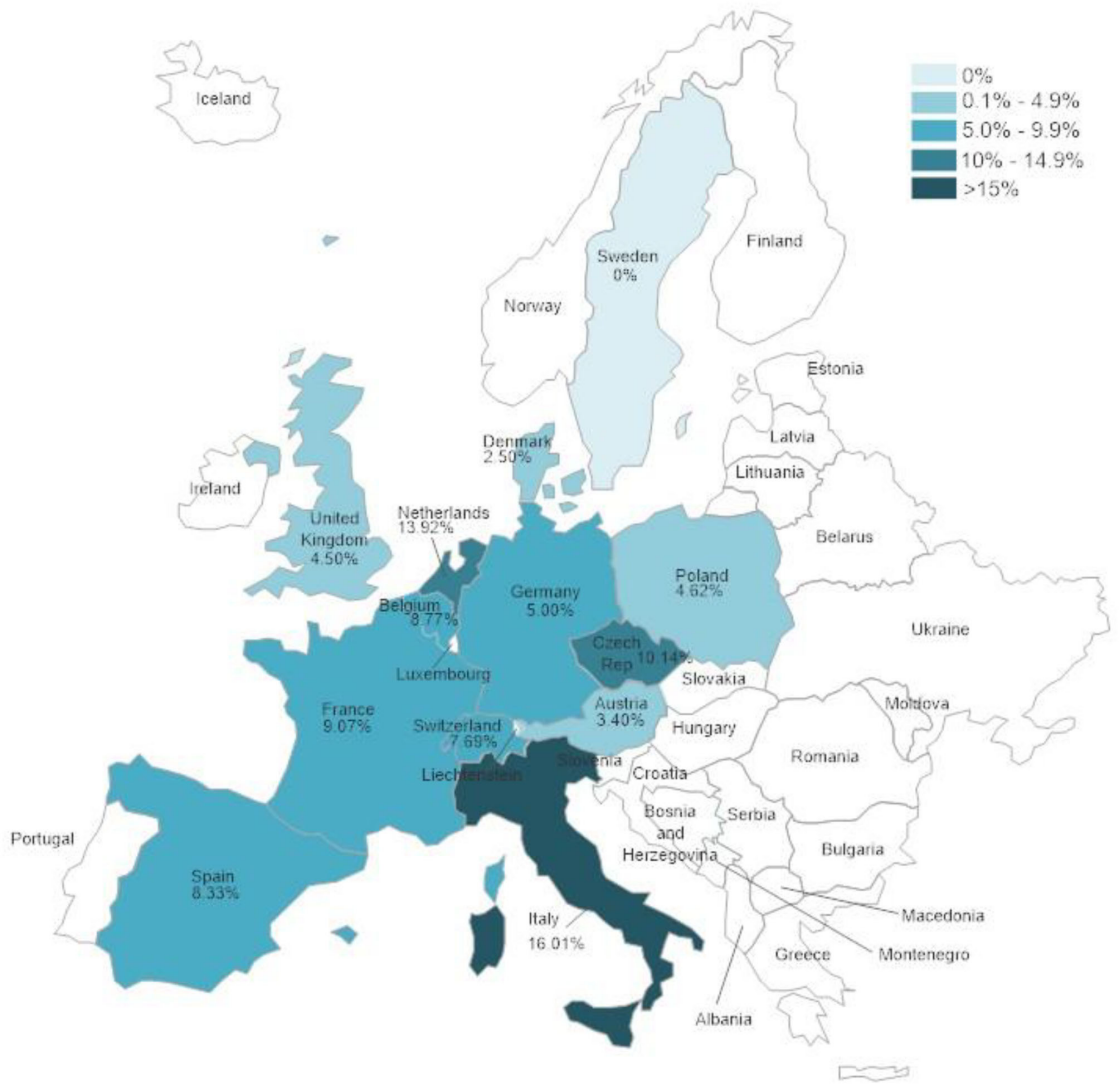
Herpesvirus positivity rates according to country in which samples were collected. Only countries from which 50 samples or more were submitted were included in the evaluation.

The tested samples originated from different countries and showed significant differences (*p* < 0.0001) in positivity rates between the different countries. Figure 1 and Supplementary Table 2 show the positivity rates in countries from which 50 samples or more were received.

### Herpesviruses in *Testudo* spp.

**Figure 2:**
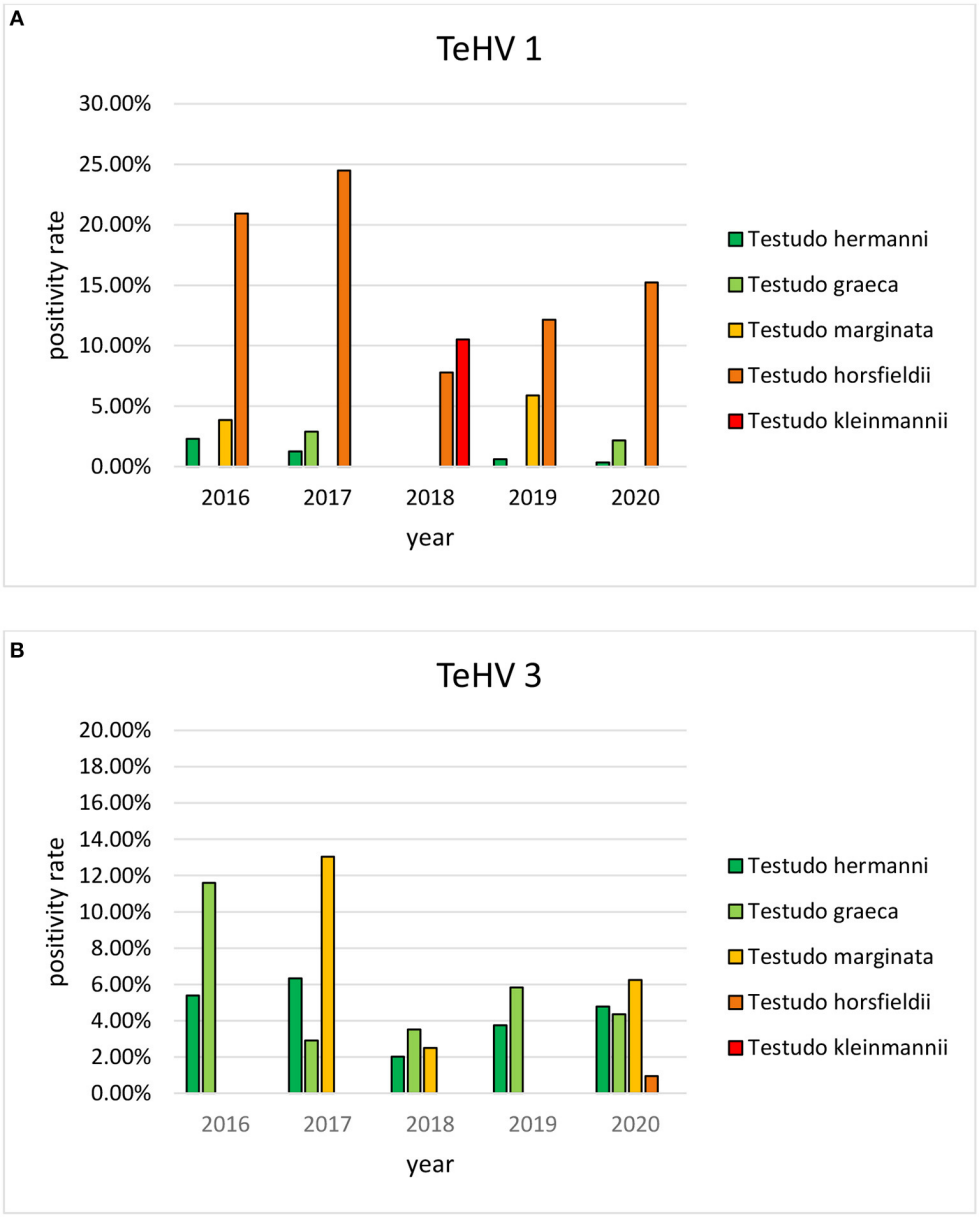
Herpesvirus positivity rates in *Testudo* species (Hermann's tortoises, *T. hermanni*, spur-thighed tortoises, *T. graeca*, marginated tortoises, *T. marginata*, Horsfield's tortoises, *T. horsfieldii*, and Egyptian tortoises, *T. kleinmanni)* for the different years of sampling (**A** TeHV 1 and **B** TeHV 3).

**Figure 3:**
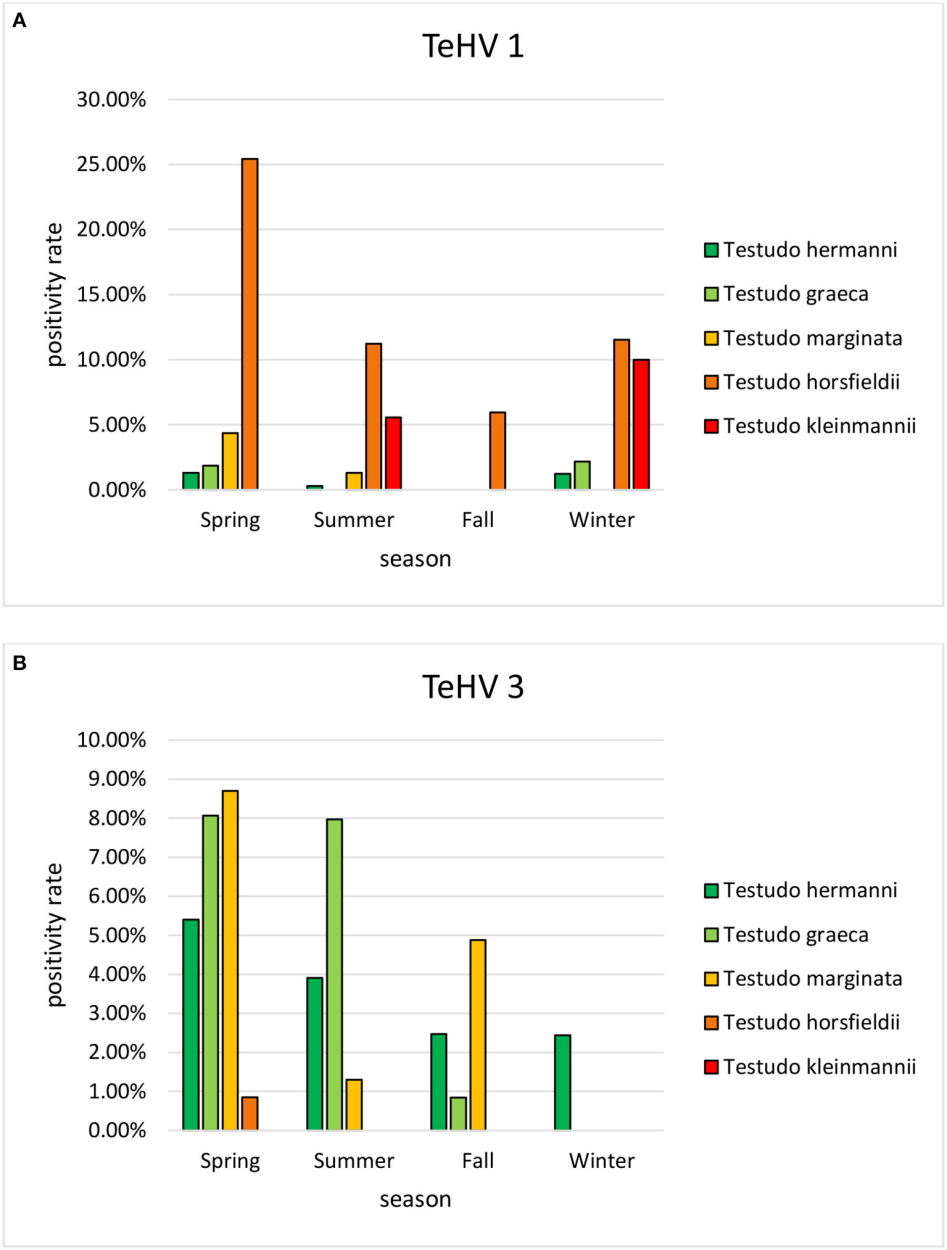
Herpesvirus positivity rates in *Testudo* species (Hermann's tortoises, *T. hermanni*, spur-thighed tortoises, *T. graeca*, marginated tortoises, *T. marginata*, Horsfield's tortoises, *T. horsfieldii*, and Egyptian tortoises, *T. kleinmanni)* according to season (**A** TeHV 1 and **B** TeHV 3).

**Figure 4:**
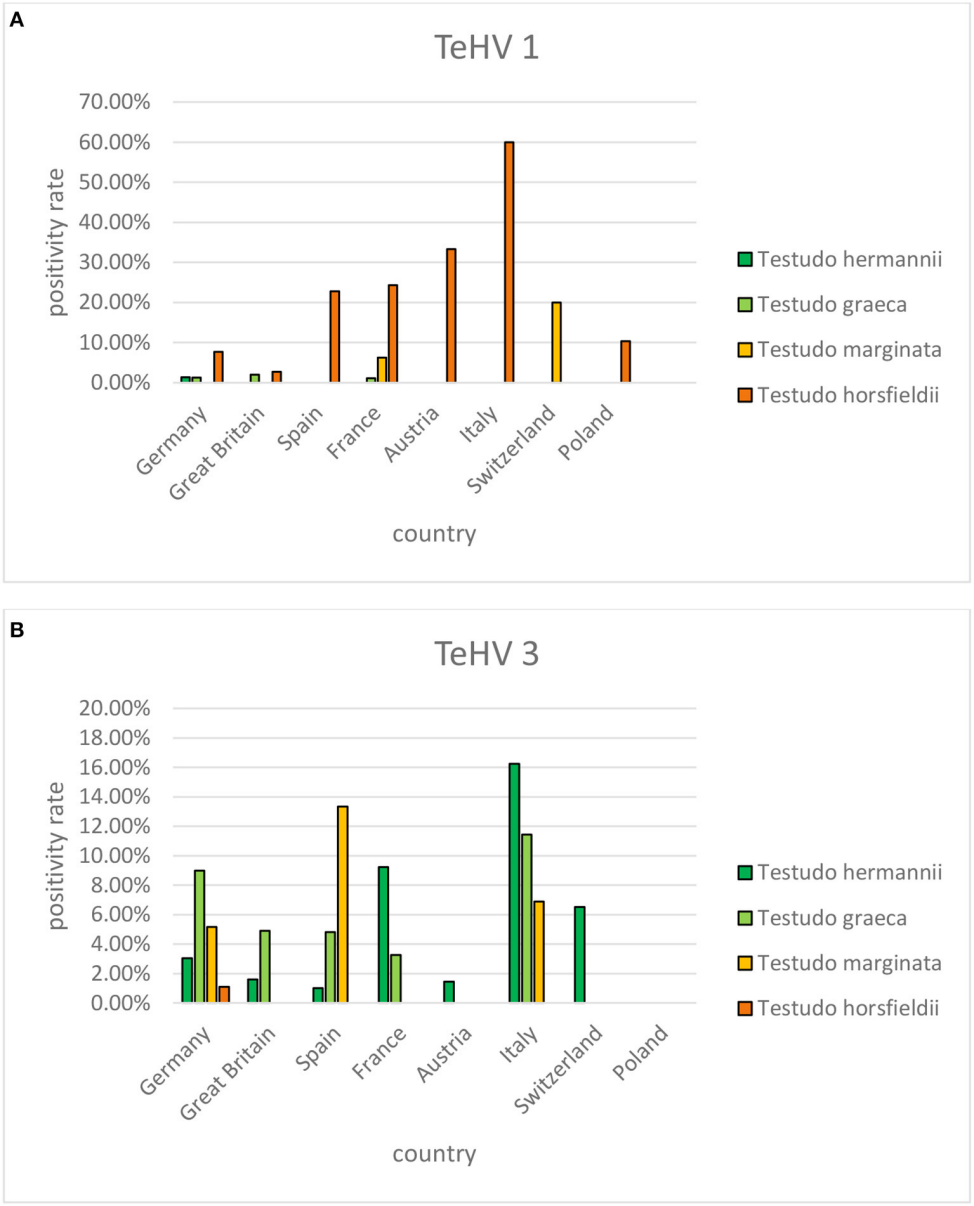
Herpesvirus positivity rates in four *Testudo* species (Hermann's tortoises, *T. hermanni*, spur-thighed tortoises, *T. graeca*, marginated tortoises, *T. marginata*, and Horsfield's tortoises, *T. horsfieldii)* according to country for countries from which more than 100 samples were submitted (**A** TeHV 1 and **B** TeHV 3).

A comparison of positivity rates in specific species showed differences among the various *Testudo* spp. tested (Hermann's tortoises, spur-thighed tortoises, marginated tortoises, *Testudo marginata*, Horsfield's tortoises, Egyptian tortoises*, Testudo kleinmanni*) (Supplementary Table 3). Herpesviruses were detected significantly more often in Horsfield's tortoises (14.96%) than in the other *Testudo* spp. tested (between 3.92 and 6.25%) (*p* < 0.0001). For all of these species, except the Egyptian tortoises (*p* = 0.1341), the positivity rate was significantly impacted by the year (*p* < 0.0001) with the highest rates measured in 2017 (11.36%) and the lowest measured in 2018 (3.45%) (Figure 2). Positivity rates also differed significantly depending on the season (*p* < 0.0001 for Hermann', spur-thighed, and Horsfield's tortoises, *p* = 0.0012 for marginated tortoises), except in the Egyptian tortoises (*p* = 0.1412) (Figure 3). The distribution of herpesvirus types detected also differed significantly (*p* < 0.0001) depending on the host species among the *Testudo* spp. tested. In Hermann's tortoises, TeHV3 (86.0%) was significantly more commonly detected than TeHV1 (14.0%). A similar distribution was found in spur-thighed tortoises (TeHV3 86.2% and TeHV1 13.8%) and marginated tortoises (TeHV3 70.0%, TeHV1 30.0%). In contrast, TeHV1 (98.15%) was more common than TeHV3 (1.85%) in Horsfield's and in Egyptian tortoises (TeHV1 100%). The distribution of TeHV1 and TeHV3 in four of the *Testudo* ssp. tested also differed significantly (*p* = 0.0002) between the eight countries from which the largest numbers of samples were tested (Figure 4). The data from the Egyptian tortoises were excluded from these analyses because only one of the 10 samples from this species from Spain and the only sample from this species from Italy were TeHV1 positive. The highest positivity rate for both TeHV1 and TeHV3 was found in samples submitted from Italy, while the lowest rates were found in Austria and Great Britain for TeHV1 and in Austria for TeHV3 (Figure 4).

### Herpesviruses in Emydidae

A comparison of positivity rates among samples from turtles in the family Emydidae also showed significant differences (*p* < 0.0001) depending on the host species (Table 1). A comparison of the three genera in the Emydidae in which herpesviruses were detected (*Emys, Terrapene*, and *Trachemys*) showed a significantly (*p* = 0.0002) higher positivity rate in box turtles (*Terrapene* spp.) than in sliders (*Trachemys* spp.) or European pond turtles (*Emys* spp.). Similar to the *Testudo* species, herpesvirus detection in Emydidae varied significantly (*p* = 0.0009) over the test period, with the positivity rate increasing from 2016 (3 of 53; 5.66%; 95% CI 1.94–15.37%) over 2017 (1 of 16; 6.25%; 95% CI 1.11–28.33%) to 2018 (5 of 37; 13.51%; 95% CI 5.91–27.97%), while no herpesviruses were detected in any of the samples tested in 2019 (*n* = 36; 0%; 95% CI 0–9.64%) and only one of 34 samples tested in 2020 was positive (2.94%; 95% CI 0.52–14.91%). A significant (*p* = 0.0065) seasonal variation was also noticed with no positive samples in spring (*n* = 36 tested; 0%; 95% CI 0–9.64%); 5 of 51 positive in summer (9.80%; 95% CI 4.26–20.97%), 4 of 67 positive in fall (5.97%; 95% CI 2.35–14.37%) and only one of 22 positive in the winter season (4.55%; 95% CI 0.81–21.80%).

## Discussion

Herpesviruses have frequently been described in various chelonian species and have been shown to be important pathogens in many of these animals (3). Previous studies screening samples from captive chelonians in Europe have reported positivity rates of 25% in various species of tortoises in Spain (19), 17% in mixed species from various collections in Belgium (18), 13.7% in captive tortoises from several European countries (17), 8.2% in various tortoise species in the United Kingdom (20), and 8.0% in a wide variety of turtle and tortoise species in various countries (13). The highest positivity rate was in tortoises specifically showing clinical signs considered typical of herpesvirus infection (19), but the other reports included both clinically ill and inapparently infected animals. The overall positivity rate reported here of 6.50% is lower than these previously reported numbers. However, the results of this study indicate that positivity rate may depend on year, season, and host species, so differences in the testing period and the range of species included influence the overall herpesvirus positivity rate. This is the first report describing herpesvirus detection in captive chelonians over a period of several years and in such a large number of samples. It is interesting to note that detection varied over the course of the 5 year period examined, with an apparent undulating pattern. Other studies reporting on prevalence of herpesvirus infections in groups or populations of chelonians have also found differences in positivity rates between individual years (32, 44), although no previous studies have stretched over more than two separate years. A factor in the numbers of positive samples detected per year could be the numbers of samples tested. This increased from 2016 and 2017 (681 resp. 581 samples) to 2018 through 2020, with over 1,000 samples per year. Reasons for this increase are not known, but changes in willingness of owners to test animals with or without clinical signs of disease could influence positivity rates. Additional study is necessary to better understand possible temporal effects on herpesvirus infection and shedding in captive chelonians. This is likely a complex issue involving multiple factors including host species, virus strain, breeding practices, and the pet trade, as well as possible additional factors such as weather, husbandry, and additional infectious agents.

The types of herpesviruses detected during the course of this study included mostly previously described types, including the two most commonly described in tortoises, TeHV1 and TeHV3 (3, 13) as well as the type most often reported in box turtles, Terrapene herpesvirus 1 (30–32, 44). In addition, this includes the first report of a TeHV2 from a tortoise in Europe. This virus has previously only been described in *Gopherus* spp. in North America (14, 45). The single TeHV2 detected was also found in a tortoise in the same genus (a Texas tortoise, *Gopherus berlandieri*) kept in Spain. This is a reminder of the possible role of the pet trade in the transport of pathogens throughout the world, as it is most likely that the virus was imported into Europe with a tortoise host. TeHV4 was also detected in a total of three tortoises tested in this study, two leopard tortoises and one tortoise of unknown species. This strain has previously been described in a leopard tortoise in Europe (16) and in a Bowsprit tortoise in the USA (15). It has been hypothesized that it may be specific to African tortoises (16, 46). This hypothesis is supported by the findings in the present study. Trachemys herpesvirus 1, which was detected in one red- and one yellow-eared (*Trachemys scripta scripta*) slider in the present study, has previously been described in a free-ranging red-eared slider in the United States (27). As for TeHV2, this is another example of international movement of viruses with their hosts, although in both cases there was no evidence from our findings of transmission of these two viruses from their presumed original North American host species into European species.

In addition to the previously described herpesviruses found in this study, three previously undescribed herpesviruses were identified in individual cases. While two of these, from a European pond turtle and a Chinese stripe-necked turtle, clustered with previously described putative members of the genus *Scutavirus*, one, from a Siebenrock's snake-necked turtle, did not. This virus was detected in a turtle from a pet store. Since the herpesvirus detected in that animal was from an oral swab and clustered with a herpesvirus from a squamate reptile (a chameleon), it is unclear whether it represents a real infection in the turtle or a possible contaminant from e.g., another reptile in the pet store. Unfortunately, the animal was lost to follow up, but the question of whether herpesviruses from reptiles in orders other than Testudines might be able to also infect chelonians requires further study. The finding of previously undescribed scutaviruses in individual cases in this study is not unexpected. As of December 2020 there were 361 species of Testudines listed in the reptile database (http://www.reptile-database.org/db-info/SpeciesStat.html). Since viruses in the genus *Scutavirus* have been hypothesized to have co-evolved with chelonian hosts, it is likely that there are many more chelonian herpesviruses yet to be discovered (1, 26).

The majority of the samples examined in this study were from tortoises in the genus *Testudo*. The distribution of herpesvirus strains found in these animals confirmed some of the previously reported data regarding species specificity of TeHV1 and 3, with TeHV1 found much more commonly in Horsfield's tortoises than in most other *Testudo* spp., while TeHV3 was more common in Hermann's, spur-thighed, and marginated tortoises. TeHV1 was, however, also found in a variety of other species, including Hermann's, spur-thighed, marginated, Egyptian, African spurred (*Centrochelys sulcata*), leopard (*Stigmochelys pardalis*), Argentine (*Chelonoidis chilensis*), and an Indian star (*Geochelone elegans*) tortoise. This extends the list of species in which this virus has been detected (3) by several species, including the marginated, African spurred, Argentine, Egyptian, and Indian star tortoises. TeHV3 was also detected in a wide variety of species in addition to the *Testudo* spp. listed above, including Horsfield's, African spurred, radiated (*Astrochelys radiata*), leopard, and a red-footed tortoise (*Chelonoidis carbonarius*). Although there were clear differences in the species in which each of these viruses was most often found, the wide range of hosts emphasizes the ability of each of these viruses to infect a wide range of tortoises in the family Testudinidae. Since the animals tested in this study were captive, findings of specific virus strains in various species also reflect circulation of strains in various holdings.

Among the non-*Testudo* tortoise species tested, there were also clear differences in types of viruses found in different species as well as in positivity rates (Table 1). Although there were smaller numbers tested in each of these compared to the *Testudo* spp., there were some interesting findings. Of the 121 radiated tortoises tested, 17 (14.05%) were positive, all with TeHV3. A similar high infection rate was reported in a previous study, but with a smaller number of animals tested (13). Whether this represents an increased susceptibility of this species for this virus or sampling bias deserves further study. Among tortoises in the genus *Chelonoidis* (Table 1), highly significant differences (*p* < 0.0001) were found in overall positivity and for the detected virus types between the different species, with a remarkably high positivity rate in Argentine tortoises (28.57%, 90% of which were TeHV1) compared to all of the other *Chelonoidis* spp. tested. In the Emydidae tested, box turtles were significantly more often herpesvirus positive than other turtles. Similar or higher positivity rates have been reported in studies of wild box turtles in the USA (32, 44).

There were significant differences between positivity rates in samples from different European countries. It is expected that trade in animals between these countries would lead to similar disease trends in all of them. The volume of trade in reptiles has been shown to be high in Europe (47–49) but it is difficult to determine differences between different countries within Europe. The country with the highest herpesvirus positivity rate, Italy, also had the highest percentages of both TeHV1 and TeHV3 detections when taken individually. A factor that could influence the numbers of herpesvirus positive results is the distribution of species kept in captivity within the different countries. However, data on the species distribution in captivity was not available. In a study screening reptiles presented to specialized veterinarians within Poland for infectious diseases, Horsfield's tortoises were the chelonian species most often seen (50). In that study, no herpesviruses were found in any of the tortoises tested. However, a possible bias toward Horsfield's tortoises over other species as pets in Poland could explain the detection of TeHV1 but no other testudinid herpesvirus types in samples from that country.

Season was shown to affect the positivity rate of herpesviruses in general as well as of TeHV1 and TeHV3 individually and for herpesviruses in Emydidae. Herpesviruses in general, as well as both TeHV1 and TeHV3 were all most often detected in samples submitted in the spring. Increased virus detection and increased herpesvirus-associated disease in tortoises in the spring has been described before (13, 41). The influence of season on susceptibility to infection, recrudescence of infection, and disease development is not yet understood, but several authors have hypothesized that seasonal differences in behavior as well as environmental temperature could influence all of these elements. The majority of species sampled in this study hibernate in the winter. Hibernation is associated with various changes in the immune system (51), and has been hypothesized to increase herpesvirus recrudescence (41). Studies screening wild-caught Blanding's turtles (*Emydoidea blandingii*) in North America for Emydoidea herpesvirus 1 have shown higher positivity rates in May than in other months (52, 53). The authors pointed out that this corresponded to the start of nesting season and increased viral shedding could be associated with the corresponding increased physiologic demands. A study of an outbreak of TeHV3 infection and associated disease in a breeding facility with a mixed collection of turtles and tortoises in Italy showed that the disease outbreak began in spring, although it persisted over several months (41). Surviving animals were most likely to shed virus in the spring, post hibernation. In contrast to the findings in tortoises, herpesviruses in turtles in the family Emydidae were most often found in the summer, followed by the fall. This corresponds to findings in free-ranging Eastern box turtles in the USA. In one such study, the highest prevalence of Terrapene herpesvirus 1 was found in July compared to September and May (44). In that case, the authors discussed increased activity and differences in behaviors, including a possible increase in contacts between animals during foraging, as possible reasons for increased detection rates in the summer. Another study screening eastern box turtles in the same states in the USA found the highest prevalence of Terrapene herpesvirus 1 in the fall (32). Influences of seasonality of herpesvirus infections may therefore be complex and requires further study.

The vast majority of samples included in this study were oral swabs. Oral swabs have been shown to be a good and relatively uninvasive sample for the detection of herpesviruses in tortoises (54). In aquatic turtles, oral swabs (28, 30, 44) or combined oral and cloacal swabs (26, 52, 53) have most often been used for the detection of herpesviruses in live animals. However, it is possible that alternative samples could have been more appropriate in some cases. The quality of sample collection was also not standardized in this study. Samples were submitted in almost all cases by veterinarians, but it is likely that there were differences in sampling technique between the different individuals that submitted samples for testing. The time between sample collection and arrival in the laboratory was also not standardized. These factors could have contributed to false negative results in some cases, so that the actual infection rate among the sampled chelonians could have been higher than detected.

The methods used for herpesvirus detection have all been described previously for use in the detection of herpesviruses in chelonians (13). The pan-herpesvirus PCR described by VanDevanter et al. (36) specifically has been very useful in detecting previously unknown herpesviruses in multiple species (9, 15, 24–26, 30) and was key to the identification of several of the viruses described here. In many cases, more specific and often more sensitive PCRs have been developed for the detection of individual herpesvirus strains in specific species and populations (28, 39, 40, 55, 56). However, these methods are not expected to be able to detect strains other than their target strains, limiting their use in the situation described here in which samples from a very wide range of species were submitted and where in numerous cases the exact species from which the sample was obtained was not identified to the laboratory.

Unfortunately, there were only very few cases in which more in-depth data on the animals from which the samples were collected was available. It was therefore not possible to evaluate the results based on clinical signs reported in the animals or on other characteristics such as sex or age of the animals tested.

It is important to note that the sample population examined in this study was biased as it relied on samples submitted by veterinarians or owners willing to pay for diagnostic testing. It is likely that testing was carried out more often in animals that were clinically ill or had known contact with clinically ill animals. The reported herpesvirus positivity rates therefore likely do not reflect the actual prevalence among captive chelonians in Europe. This bias is likely to lead to a higher estimated prevalence of herpesvirus infection than the actual prevalence. However, it is also important to note that diagnosis in this study relied on PCR detection, so that in some cases, latently infected animals not actively shedding virus might have tested false negative. The numbers of latently infected animals that would not have tested positive using the methods described in this study is not known, but likely depends on various factors, especially host species and virus strain.

The results of this study provide important information on both the diversity and relative importance of various herpesvirus strains in a wide range of captive chelonian species as well as some information on variation in prevalence of infection and shedding depending on a number of different factors. It is important to continue to monitor herpesvirus positivity rates and herpesvirus strains in captive reptiles over time to better understand their importance and impacts on reptiles in captivity. Additional studies on herpesviruses in the reptile trade as well as interactions between infections in captive reptiles, reptiles in trade, and wild reptiles would be of interest.

## Data Availability Statement

The raw data supporting the conclusions of this article will be made available by the authors, without undue reservation.

## Author Contributions

CL participated in the conceptualization, compiled, summarized, and analyzed the data and drafted figures and tables. EM participated in the conceptualization and planning of the project. RM participated in the conceptualization of the project and data analysis and led manuscript development. All authors provided critical feedback and contributed to the final manuscript.

## Conflict of Interest

CL, EM, and RM are employed by a private laboratory that offers diagnostic testing for veterinarians. This employment did not influence the study design, data interpretation, or writing of this manuscript.

## Publisher's Note

All claims expressed in this article are solely those of the authors and do not necessarily represent those of their affiliated organizations, or those of the publisher, the editors and the reviewers. Any product that may be evaluated in this article, or claim that may be made by its manufacturer, is not guaranteed or endorsed by the publisher.
