# Supplementary material for: Herpesviruses in Captive Chelonians in Europe Between 2016 and 2020
Source: Front Vet Sci. 2021 Oct 13;8:733299. doi: 10.3389/fvets.2021.733299 (PMC8549816; doi:10.3389/fvets.2021.733299)
Supplement: Supplementary file 1 [file Table_1.DOCX]

**Supplementary Table 1**: Sequenences of interest from this paper

| Laboratory number | Host species | Sequence length (bp) | Closest match via BLAST | Identity | Nt sequence |
| --- | --- | --- | --- | --- | --- |
| 1709M05477 | *Gopherus berlandieri* | 142 | TeHV2 (AY916792.1) | 100% | GGCGATGGGTTTATTACCGTGTTTGGAAGTTGCAGCTACCGTCACCACGGTGGGGCGCAATATGCTGTTGGCCACCCGCGATTACATACACGACAGATGGGACGAGAGAGAAAAGTTTTTGGCCGATTTTCCTCAATTTGCT |
| 1812R18272 | *Stigmochelys pardalis* | 139 | TeHV4 (GQ222415.1) | 100% | GTTGCTATGGGTCTTTTGCCGTGCTTGGAAGTAGCTGCCACAGTAACCACTGTTGGGCGCAATATGTTGTTGTCCACCAGGGATTATATCCACGAAAGATGGTCGGATAGGGAACAATTTCTGGCGGATTTTCCTCAAA |
| 1602S63268 | *Stigmochelys pardalis* | 119 | TeHV4 (GQ222415.1) | 97.5% | GTGGCTATGGGTCTTTTGCCGTGCTTAGAAGTAGCTGCCACAGTAACCACTGTTGGGCGCAATATGTTGTTGTCCACCAGGGATTATATCCACGAAAGATGGTCGGATAGGGAAAAATT |
| 1603S11728 | Spezies unknown | 116 | TeHV4 (GQ222415.1) | 98.3% | GTGGCTATGGGTCTTTTGCCGTGCTTAGAAGTAGCTGCCACAGTAACCACTGTTGGGCGCAATATGTTGTTGTCCACCAGGGATTATATCCACGAAAGATGGTCGGATAGGGAAAA |
| 1908T27277 | *Mauremys sinensis* | 161 | HV isolate from a *Pelusios williamsi* (KX374559.1) | 76.4% | TGTAACTCGGTGTAtGGgTTtACgGGtGTGGCTTCTGGTTTATTGCCGTGCCTGGAGGTGGCCGCCACCGTCACCACCCTCGGGCGACAAATGTTACTCGACACGCGCGATTACATTCACGCCAGGTGGACGTCCGCCGCGGCGCTCTtgGCCGATTTCCC |
| 2011S00660 | *Emys orbicularis* | 180 | Terrapene HV 1 (KJ004665.1) | 94.4% | GCCATGGGCCTGCTGCCGTGTCTGGAGGTGGCCGCCACCGTCACCACCGTTGGGCGCAATATGCTGTTGGCCACCCGAGATTATATCCACGCCCATTGGAGCGAGCGGGAAAAGTTTCTGGCCGACTTCCCGGAGCTCGGCGCTCACGTGATCCCAGACGAGCACCACTCCCTGCGGATA |
| 1803T62630 | *Chelodina rugosa* | 137 | Chamaeleonid herpesvirus 1 (MW015088.1) | 100% | GGCCTACGGCCTACTTCCGTGTCTGGAGGTCGCCGCCACCGTCACTACCCTGGGGCGGACCATGTTGGACGCCACCAAACGGTTCATCGAGGGGCGGTGGGGCGCGGACCTGTCCCGTCTGACGGGGGACTTCCCCG |

**Supplementary Table 2:** Herpesvirus positivity rate according to virus strain and country of sample origin for countries from which more than 50 samples were submitted. Results shown as: Number positive (% positive from the individual country; 95% confidence interval (CI)).

| Country | Total (n) | Herpesvirus positive | TeHV 1 | TeHV 2 | TeHV 3 | TeHV 4 | Terrapene HV 1 | Trachemys Herpesvirus 1 | Other Alphaherpesviruses |
| --- | --- | --- | --- | --- | --- | --- | --- | --- | --- |
| Germany | 1421 | 71 (5.0%; CI 3.98-6.26%) | 25 (1.76%; CI 0.12-2.59%) | 0 (0%; CI 0-0.27%) | 45 (3.17%; CI 2.38-4.21%) | 0 (0%; CI 0-0.27%) | 1 (0.07%; CI 0.01-0.04%) | 0 (0%; CI 0-0.27) | 0 (0%; CI 0-0.27%) |
| Great Britain | 999 | 45 (4.50%; CI 3.38-5.97%) | 22 (2.20%; CI 1.46-3.31%) | 0 (0%; CI 0-0.38%) | 22 (2.20%; CI 1.46-3.31%) | 1 (0.10%; CI 0.02-0.56%) | 0 (0%; 0-0.38%) | 0 (0%; 0-0.38%) | 0 (0%; CI 0-0.38%) |
| Spain | 648 | 54 (8.33%; CI 6.44-10.71%) | 31 (4.78%; CI 3.39-6.71%) | 1 (0.15%; CI 0.03-0.86%) | 16 (2.47%; CI 1.53-3.97%) | 0 (0%; CI 0-0.59%) | 6 (0.93%; CI 0.43-2.01%) | 0 (0%; CI 0-0.59%) | 0 (0%; CI 0-0.59%) |
| France | 441 | 40 (9.07%; CI 6.73-12.12%) | 21 (4.76%; CI 3.13-7.17%) | 0 (0%; CI 0-0.86%) | 17 (3.85%; CI 2.42-6.08%) | 1 (0.23%; CI 0.04-1.28%) | 0 (0%; CI 0-0.86%) | 0 (0%; CI 0-0.86%) | 1 (0.23%; CI 0.04-1.28%) |
| Austria | 294 | 10 (3.40%; CI 1.86-6.15%) | 4 (1.36%; CI 0.53-3.44%) | 0 (0%; CI 0-1.29%) | 6 (2.04%; 0.94-4.38%) | 0 (0%; CI 0-1.29%) | 0 (0%; CI 0-1.29%) | 0 (0%; CI 0-1.29%) | 0 (0%; CI 0-1.29%) |
| Italy | 281 | 45 (16.01%; CI 12.19-20.75%) | 15 (5.34%; CI 3.26-8.62%) | 0 (0%; 0-1.35%) | 28 (9.96%; CI 6.98-14.02%) | 0 (0%; CI 0-1.35%) | 0 (0%; CI 0-1.35%) | 0 (0%; CI 0-1.35%) | 2 (0.71%; CI 0.19-2.55%) |
| Switzerland | 143 | 11 (7.69%; CI 4.35-13.25%) | 3 (2.10%; CI 0.72-5.99%) | 0 (0%, CI 0-2.62%) | 8 (5.59%; CI 2.86-10.64%) | 0 (0%; CI 0-2.62%) | 0 (0%; CI 0-2.62%) | 0 (0%; CI 0-2.62%) | 0 (0%; CI 0-2.62%) |
| Poland | 130 | 6 (4.62%; CI 2.14-9.71%) | 4 (3.08%; CI 1.20-7.65%) | 0 (0%; CI 0-2.87%) | 0 (0%; CI 0-2.87%) | 0 (0%; CI 0-2.87%) | 0 (0%; CI 0-2.87%) | 2 (1.54%; CI 0.42-5.44%) | 0 (0%; CI 0-2.87%) |
| Denmark | 80 | 2 (2.50%; CI 0.69-8.66%) | 0 (0%; CI 0-4.58%) | 0 (0%; CI 0-4.58%) | 2 (2.50%; CI 0.69-8.66%) | 0 (0%; CI 0-4.58%) | 0 (0%; CI 0-4.58%) | 0 (0%; CI 0-4.58%) | 0 (0%; CI 0-4.58%) |
| Netherlands | 79 | 11 (13.92%; CI 7.95-23.23%) | 9 (11.39%; CI 6.11-20.25%) | 0 (0%; CI 0-4.64%) | 2 (2.53%; CI 0.70-8.77%) | 0 (0%; CI 0-4.64%) | 0 (0%; CI 0-4.64%) | 0 (0%; CI 0-4.64%) | 0 (0%; CI 0-4.64%) |
| Czech Republic | 69 | 7 (10.14%; CI 5.0-19.49%) | 4 (5.80%; CI 2.28-13.98%) | 0 (0%; CI 0-5.27%) | 2 (2.90%; CI 0.80-9.97%) | 1 (1.45%; CI 0.26-7.76%) | 0 (0%; CI 0-5.27%) | 0 (0%; CI 0-5.27%) | 0 (0%, CI 0-5.27%) |
| Belgium | 57 | 5 (8.77%; CI 0.38-18.94%) | 4 (7.02%; CI 2.76-16.70%) | 0 (0%; CI 0-6.31%) | 1 (1.75%; CI 0.31-9.28%) | 0 (0%; CI 0-6.31%) | 0 (0%; CI 0-6.31%) | 0 (0%; CI 0-6.31%) | 0 (0%; CI 0-6.31%) |
| Sweden | 51 | 0 (0%; CI 0-7.0%) | 0 (0%; CI 0-7.0%) | 0 (0%; CI 0-7.0%) | 0 (0%; CI 0-7.0%) | 0 (0%; CI 0-7.0%) | 0 (0%; CI 0-7.0%) | 0 (0%; CI 0-7.0%) | 0 (0%; CI 0-7.0%) |

**Supplementary Table** **3:** Herpesvirus positivity rates in *Testudo* species (Hermann’s tortoises, *T. hermanni*, spur-thighed tortoises, *T. graeca*, marginated tortoises, *T. marginata*, Horsfield’s tortoises, *T. horsfieldii*, and Egyptian tortoises, *T. kleinmanni*) according to year and season of sample collection and country of sample origin. Results shown as: Number (% of samples from that species submitted in the specific year, season, or from that specific country; 95% confidence interval (CI)).

|  | *Testudo hermanni* | | | *Testudo graeca* | | | *Testudo marginata* | | | *Testudo horsfieldii* | | | *Testudo kleinmannii* | | |
| --- | --- | --- | --- | --- | --- | --- | --- | --- | --- | --- | --- | --- | --- | --- | --- |
| Year | Neg. | TeHV 1 | TeHV3 | Neg. | TeHV 1 | TeHV 3 | Neg. | TeHV 1 | TeHV 3 | Neg. | TeHV 1 | TeHV 3 | Neg. | TeHV 1 | TeHV 3 |
| 2016 | 120 (92.31%; CI 86.42-95.77%) | 3 (2.31%; CI 0.79-6.57%) | 7 (5.38%; CI 2.63-10.69%) | 61 (88.41%; CI 78.76-94.01%) | 0 (0%; CI 0-5.27%) | 8 (11.59%; CI 5.99-21.24%) | 25 (96.15%; CI 81.10-99.80%) | 1 (3.85%; CI 0.68-18.90%) | 0 (0%; CI 0-12.87%) | 34 (79.07%; CI 64.79-88.58%) | 9 (20.93%; CI 11.42-35.21%) | 0 (0%; CI 0-8.20%) | 1 (100%, CI 20.65-100%) | 0 (0%; CI 0-79.35%) | 0 (0%; 0-79.35%) |
| 2017 | 73 (92.41%; CI 84.41-96.48%) | 1 (1.27%; CI 0.23-6.83%) | 5 (6.33%; CI 2.73-13.98%) | 65 (94.20%; CI 86.02-97.72%) | 2 (2.90%; CI 0.08-9.97%) | 2 (2.90%; CI 0.08-9.97%) | 20 (86.96%; CI 67.88-95.46%) | 0 (0%; CI 0-14.31%) | 3 (13.04%; CI 4.54-32.12%) | 37 (75.51%; CI 61.91-85.40% | 12 (24.49%; CI 14.60-38.09%) | 0 (0%; CI 0-7.27%) | 2 (100%; CI 34.24-100%) | 0 (0%; 0-65.76%) | 0 (0%; CI 0-65.76%) |
| 2018 | 244 (97.99%; CI 95.38-99.14%) | 0 (0%; CI 0-1.52%) | 5 (2.01%; CI 0.86-4.62%) | 110 (96.49%; 91.32-98.63%) | 0 (0%; CI 0-3.26%) | 4 (3.51%; CI 1.37-8.68%) | 39 (97.50%; CI 87.12-99.56%) | 0 (0%; CI 0-8.76%) | 1 (2.50%; CI 0.44-12.88%) | 83 (92.22%; CI 84.80-96.18%) | 7 (7.78%; CI 3.82-15.20%) | 0 (0%; CI 0-4.09%) | 17 (89.47%; CI 68.60-97.06%) | 2 (10.53%; CI 2.94-31.40%) | 0 (0%; CI 0-16.82%) |
| 2019 | 307 (95.64%; CI 92.82-97.39) | 2 (0.62%; CI 0.17-2.24%) | 12 (3.74%; CI 2.15-6.42%) | 113 (94.17%; CI 88.45-97.15%) | 0 (0%; CI 0-0.31%) | 7 (5.83%; CI 2.85-11.55%) | 32 (94.12%; CI 80.91-98.37%) | 2 (5.88%; CI 1.63-19.09%) | 0 (0%; CI 0-10.15%) | 65 (87.84%; CI 78.48-93.47%) | 9 (12.16%; CI 6.53-21.52%) | 0 (0%; CI 0-4.93%) | 10 (100%; CI 72.25-100%) | 0 (0%; CI 0-27.75%) | 0 (0%; 0-27.75%) |
| 2020 | 278 (94.88%; CI 91.73-96.87%) | 1 (0.34%; CI 0.06-1.91%) | 14 (4.78%; CI 2.87-7.86%) | 86 (93.48%; CI 86.50-96.98%) | 2 (2.17%; CI 0.06-7.58%) | 4 (4.35%; CI 0.17-10.65%) | 45 (93.75%; CI 83.16-97.85%) | 0 (0%; CI 0-7.41%) | 3 (6.25%; CI 2.15-16.84%) | 88 (83.81%; CI 75.59-89.64%) | 16 (15.24%; CI 9.60-23.33%) | 1 (0.95%; CI 0.17-5.19%) | 19 (100%; CI 83.18-100%) | 0 (0%; 0-16.82%) | 0 (0%; 0-16.82%) |
| Season |  |  |  |  |  |  |  |  |  |  |  |  |  |  |  |
| Spring | 363 (93.32%; CI 90.39-95.40%) | 5 (1.29%; CI 0.55-2.98%) | 21 (5.40%; CI 3.56-8.11%) | 145 (90.06%; CI 84.46-93.79%) | 3 (1.86%; CI 0.63-5.33%) | 13 (8.07%; CI 4.78-13.32%) | 40 (86.96%; CI 74.34-93.88%) | 2 (4.35%; CI 1.20-14.54%) | 4 (8.70%; CI 3.44-20.33%) | 87 (73.73%; CI 65.13-80.83%) | 30 (25.42%; CI 18.42-33.96%) | 1 (0.85%; CI 0.15-4.65%) | 13 (100%; CI 77.19-100%) | 0 (0%; CI 0-22.81%) | 0 (0%; CI 0-22.81%) |
| Summer | 343 (95.81%; CI 93.20-97.44%) | 1 (0.28%; CI 0.05-1.57%) | 14 (3.91%; CI 2.34-6.46%) | 127 (92.03%; CI 86.29-95.49%) | 0 (0%; CI 0-2.71%) | 11 (7.97%; CI 4.51-13.71%) | 75 (97.40%; CI 91.01-99.28%) | 1 (1.30%; CI 0.23-7.00%) | 1 (1.30%; CI 0.23-7.00%) | 95 (88.79%; CI 81.42-93.47%) | 12 (11.21%; CI 6.53-18.58%) | 0 (0%; CI 0-3.47%) | 17 (94.44%; CI 74.24-99.01%) | 1 (5.56%; CI 0.99-25.76%) | 0 (0%; CI 0-17.59%) |
| Fall | 237 (97.53%; CI 94.72-98.86%) | 0 (0%; CI 0-1.56%) | 6 (2.47%; CI 1.14-5.28%) | 118 (99.16%; 95.39-99.85%) | 0 (0%; CI 0-3.13%) | 1 (0.84%; CI 0.15-4.61%) | 39 (95.12%; CI 83.86-98.65%) | 0 (0%; CI 0-8.57%) | 2 (4.88%; CI 1.35-16.14%) | 79 (94.05%; CI 86.81-97.43%) | 5 (5.95%; CI 2.57-13.19%) | 0 (0%; CI 0-4.37%) | 10 (100%; CI 72.25-100%) | 0 (0%; CI 0-27.75%) | 0 (0%; CI 0-27.75%) |
| Winter | 79 (96.34%; CI 89.79-98.75%) | 1 (1.22%; CI 0.22-6-59%) | 2 (2.44%; CI 0:67-8.46%) | 45 (97.83%; CI 88.67-99.62%) | 1 (2.17%; CI 0.38-11.33%) | 0 (0%; CI 0-7.71%) | 7 (100%; CI 64.57-100%) | 0 (0%; CI 0-35.43%) | 0 (0%; CI 0-35.43%) | 46 (88.46%; CI 77.03-94.60%) | 6 (11.54%; CI 5.40-22.97%) | 0 (0%; CI 0-6.88%) | 9 (90.0%; CI 59.59-98.21%) | 1 (10.0%; CI 1.79-40.41%) | 0 (0%; CI 0-27.75%) |
| Country |  |  |  |  |  |  |  |  |  |  |  |  |  |  |  |
| Germany | 504 (95.63%; CI 93.54-97.08%) | 7 (1.33%; CI 0.65-2.72%) | 16 (3.04%; CI 1.88-4.88%) | 80 (89.89%; CI 81.89-94.59%) | 1 (1.22%; CI 0.20-6.09%) | 8 (8.99%; CI 4.63-16.75%) | 55 (94.83%; CI 85.86-98.23%) | 0 (0%; CI 0-6.21%) | 3 (5.17%; CI 1.77-14.14%) | 83 (91.21%; CI 83.60-95.48%) | 7 (7.69%; CI 3.77-15.03%) | 1 (1.10%; CI 0.19-5.97%) | 7 (100%; CI 64.57-100%) | 0 (0%; 0-35.43%) | 0 (0%; CI 0-35.43%) |
| Great Britain | 61 (98.39%; CI 91.42-99.72%) | 0 (0%; CI 0-5.83%) | 1 (1.61%; CI 0.28-8.58%) | 95 (93.14%; 86.51-96.64%) | 2 (1.96%; CI 0.54-6.87%) | 5 (4.90%; CI 2.11-10.96%) | 11 (100%; CI 74.12-100%) | 0 (0%; CI 0-25.88%) | 0 (0%, 0-25.88%) | 73 (97.33%; CI 90.78-99.26%) | 2 (2.67%; CI 0.74-9.22%) | 0 (0%; CI 0-4.87%) | 12 (100%; CI 75.75-100%) | 0 (0%; CI 0-24.25%) | 0 (0%; CI 0-24.25%) |
| Spain | 97 (98.98%; CI 94.45-99.82%) | 0 (0%; CI 0-3.77%) | 1 (1.02%; CI 0.18-0.55%) | 99 (95.19%; CI 89.23-97.93%) | 0 (0%; CI 0-3.56%) | 5 (4.81%; CI 2.07-10.77%) | 13 (86.67%; CI 62.12-96.27%) | 0 (0%; CI 0-20.39%) | 2 (13.33%; CI 3.73-37.88%) | 61 (77.22%; CI 66.84-85.8%) | 18 (22.78%; CI 14.92-33.16%) | 0 (0%; CI 0-4.64%) | 10 (90.91%, CI 62.27-98.38%) | 1 (9.09%; CI 1.62-37.73%) | 0 (0%; CI 0-25.88%) |
| France | 59 (90.77%; CI 81.29-95.70%) | 0 (0%; CI 0-5.58%) | 6 (9.23%; CI 0.43-18.71%) | 88 (95.65%; CI 89.35-98.30%) | 1 (1.09%; CI 0.19-5.91%) | 3 (3.26%; CI 1.11-9.15%) | 15 (93.75%; CI 71.67-98.89%) | 1 (6.25%; CI 1.11-28.33%) | 0 (0%; CI 0-19.36%) | 28 (75.68%; CI 59.89-86.64%) | 9 (24.32%; CI 13.36-40.11%) | 0 (0%; CI 0-9.41%) | 0 (0%) | 0 (0%) | 0 (0%) |
| Austria | 136 (98.55%; CI 94.87-99.60) | 0 (0%; CI 0-2.71%) | 2 (1.45%; CI 0.04-5.13%) | 7 (100%; CI 64.57-100%) | 0 (0%; CI 0-35.43%) | 0 (0%; CI 0-35.43%) | 18 (100 %; CI 82.41-100%) | 0 (0%; CI 0-17.59%) | 0 (0%; CI 0-17.59%) | 4 (66.67%; CI 30.0-90.32%) | 2 (33.33%; CI 9.68-70.0%) | 0 (0%; CI 0-39.03%) | 1 (100%; CI 20.65-100%) | 0 (0%; CI 0-79.35%) | 0 (0%; CI 0-79.35%) |
| Italy | 67 (83.75%; CI 74.16-90.25%) | 0 (0%; 0-4.58%) | 13 (16.25%; CI 9.75-25.84%) | 31 (88.57%; CI 74.05-95.46%) | 0 (0%; CI 0-12.32) | 4 (11.43%; CI 4.54-25.95%) | 27 (93.10%; CI 78.03-98.09%) | 0 (0%; CI 0-11.70%) | 2 (6.90%; CI 1.91-21.97%) | 8 (40.0%; CI 21.88-63.59%) | 12 (60.0%; CI 38.66-78.12%) | 0 (0%; CI 0-16.11%) | 0 (0%; CI 0-79.35%) | 1 (100%; CI 20.65-100%) | 0 (0%; CI 0-79.35%) |
| Switzerland | 43 (93.48%; CI 82.50-97.76%) | 0 (0%; CI 0-7.71%) | 3 (6.52%; 2.24-17.50%) | 18 (100%; CI 82.41-100%) | 0 (0%; CI 0-17.59%) | 0 (0%; 0-17.59%) | 8 (80.00%; CI 49.02-94.33%) | 2 20.00%; CI 5.67-50.98%) | 0 (0.0%; CI 0-27.75%) | 0 (0%) | 0 (0%) | 0 (0%) | 2 (100%; CI 34.24-100%) | 0 (0%; CI 0-65.76%) | 0 (0%; CI 0-65.76%) |
| Poland | 16 (100%; CI 80.64-100%) | 0 (0%; CI 0-19.36%) | 0 (0%; CI 0-19.36%) | 2 (100%; CI 34.24-100%) | 0 (0%; 0-65.76%) | 0 (0%; CI 0-65.76%) | 0 (0.0%) | 0 (0.0%) | 0 (0.0%) | 26 (89.66%; CI 73.62-96.42%) | 3 (10.34%; CI 3.58-26.38%) | 0 (0%; CI 0-11.70%) | 2 (100%; CI 34.24-100%) | 0 (0%; CI 0-65.76%) | 0 (0%; CI 0-65.76%) |
